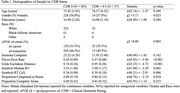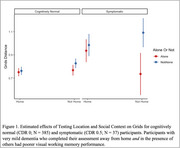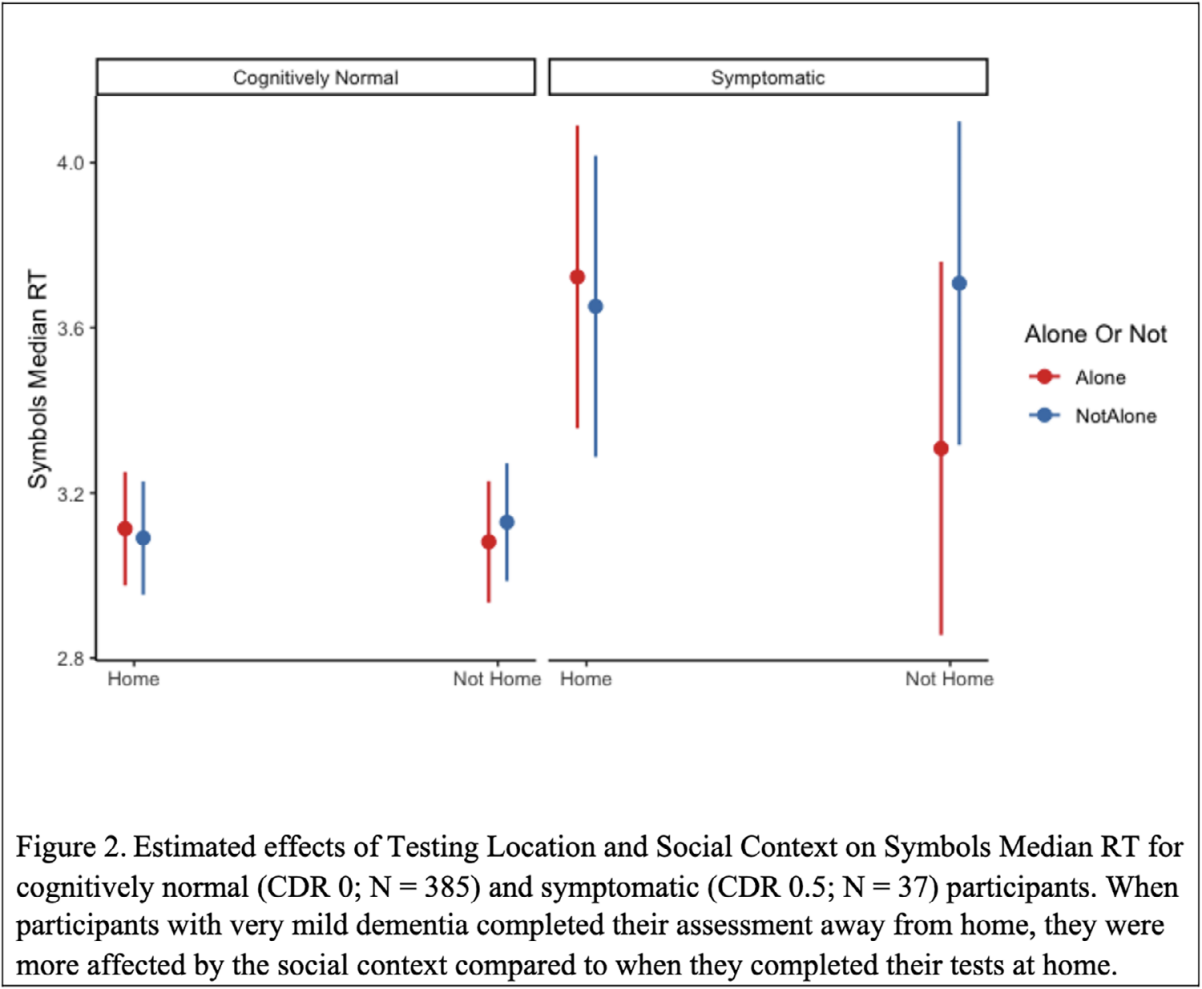# The Impact of Testing Environments on Remote Cognitive Assessments in Alzheimer's Disease

**DOI:** 10.1002/alz70857_107034

**Published:** 2025-12-26

**Authors:** Samhita Katteri, Matthew S. Welhaf, Hannah M Wilks, Andrew J. Aschenbrenner, John C. Morris, Jason J. Hassenstab

**Affiliations:** ^1^ Washington University in St. Louis, St. Louis, MO, USA; ^2^ Knight Alzheimer Disease Research Center, St. Louis, MO, USA

## Abstract

**Background:**

High‐frequency remote cognitive assessments are increasingly popular for studies of neurodegenerative disease. Because assessments are completed unsupervised and in everyday environments, there are concerns about how common distractions may impact performance and whether these distractions may differentially impact those experiencing the earliest symptoms of dementia. Using a large sample of comprehensively phenotyped older adults who completed high‐frequency cognitive assessments on their personal smartphones, we examined whether testing location, social environment, and additional reported distractions predicted cognitive performance.

**Method:**

Participants were enrolled in the Ambulatory Research in Cognition (ARC) study at the Knight Alzheimer Disease Research Center (Knight ADRC; Table 1). Participants (*N* = 422) were categorized as either cognitively normal (*N* = 385) or as having very mild dementia (*N* = 37) based on a Clinical Dementia Rating (CDR) score of 0 or 0.5, respectively. ARC is a smartphone application in which participants complete ultra brief tests of processing speed, working memory, and associate memory up to four times per day over seven consecutive days. Prior to each testing session, participants reported their current location and social surroundings, which were used to distinguish whether participants were at home or alone at the time of assessment. After each session, participants were asked if they experienced interruptions during assessments.

**Result:**

Across all participants, interactions between CDR status, testing location, and social context showed slightly worse performance on associate memory and working memory, but not processing speed. However, CDR 0.5s had slightly worse scores on working memory and processing speed when completed away from home and around others compared to their tests taken at home and alone (Figures 1 and 2). When interrupted sessions were removed (∼12% of assessments), small effects of environmental distractions on cognition remained, but only for CDR 0.5s.

**Conclusion:**

Social context and location of unsupervised remote cognitive testing has small impacts on performance, but these impacts were not consistent across cognitive domains and were mostly limited to participants demonstrating the earliest symptoms of dementia. Remote cognitive testing provides valid and reliable data, but care should be taken to allow participants to self‐report distractions during assessments.